# Work-related exposure to organic solvents and the risk for multiple sclerosis—a systematic review

**DOI:** 10.1007/s00420-020-01564-z

**Published:** 2020-09-02

**Authors:** Lars Gerhardsson, Linda Hou, Kjell Pettersson

**Affiliations:** 1 Department of Occupational and Environmental Medicine, School of Public Health and Community Medicine, Institute of Medicine, Sahlgrenska Academy, University of Gothenburg, Medicinaregatan 16A, Box 414, 405 30 Gothenburg, Sweden; 2 Institute of Neuroscience and Physiology, Rehabilitation Medicine, Sahlgrenska Academy, University of Gothenburg, Gothenburg, Sweden; 3 Feelgood Occupational Health Care AB, Gothenburg City, Sweden

**Keywords:** Organic solvents, Occupational exposure, Multiple sclerosis, Meta-analysis

## Abstract

**Purpose:**

Multiple sclerosis (MS) is a chronic progressive neurological disorder. Several environmental factors have been discussed as possible causing agents, e.g. organic solvents, whose impact on the disease is analysed in this review.

**Methods:**

Systematic search strategies were used to identify high-quality studies of workers exposed to organic solvents, published up to September 30, 2019, in databases, such as PubMed, Cochrane library and Scopus. The exposure was in most studies obtained by questionnaires, supplemented with telephone interviews. The diagnosis MS was mainly detemined following a thorough neurological examination. Finally, fourteen case–control studies and two cohort studies met the inclusion criteria and were included in the meta-analysis. Random effects models were used to pool the results of the studies.

**Results:**

The odds ratios from the 14 case–control studies included in the meta-analysis ranged from 0.12–4.0. Five case–control studies and one cohort study showed a significant association between the development of multiple sclerosis and exposure to organic solvents. The results from the other nine case–control studies and from one of the two cohort studies did not reach statistical significance. The pooled data from the 14 case–control studies gave an OR of 1.44 (95% CI 1.03–1.99), which shows a moderately increased risk of developing MS after exposure to organic solvents.

**Conclusions:**

The final interpretation of the result is that organic solvents may be slightly associated with an increased risk to develop MS. In addition, other factors, e.g. genetic markers and smoking, may contribute to the development of the disease.

## Introduction

Multiple sclerosis (MS) is a demyelinating disease, which is found in all parts of the world. The prevalence varies greatly, from high levels in North America and Europe (> 100/100,000 inhabitants) to low rates in Eastern Asia and the sub-Saharan Africa (2/100,000 population) (Leray et al. 2016). During the first 20 years of the disease progress, the mortality is similar in patients with MS and in referents. However, as the disease deteriorates, life expectancy is reduced by 6–7 years in MS patients.

Exposure to Epstein-Barr virus is a well-known risk factor for the development of MS, particularly if it is symptomatic and arises after childhood. In addition, smoking and low vitamin D levels in blood have been reported to increase the risk of developing MS as well as age, as the incidence increases, as the subjects get older. From a genetic point of view, the association between HLA-DRB1*15:01 and a high risk of MS has been known for decades. More recently, also other immunogenetic markers, such as IL2RA and IL7RA, have been identified. At present more than 100 genetic variants influencing the development of MS have been reported in studies based on genome-wide associations. Most of them are involved in the immune response and are often associated with other autoimmune diseases.

MS is not only characterized by physical disabilities, such as weakness and walk and balance difficulties, but also by cognitive impairment. About 40–65% of MS patients may have cognitive dysfunctions of different types (Amato et al. 2008). The quality of life is reduced by these physical and cognitive dysfunctions, which contribute to work disability and sick leave, thereby increasing the economic burden, both for the patient and society.

It is important to identify possible risk factors, e.g. organic solvents, to reduce the risk of contracting MS. Several reviews dealing with this topic have been published (Barragan-Martinez et al. 2012; Cooper et al. 2002; Landtblom 1997; Landtblom et al. 1996; Marrie 2004), based on original papers published up to 2012. Some of them indicated an increased risk for developing MS after exposure to organic solvents. However, some of them also included exposure to anaesthetic gases, which makes the interpretation of the results more difficult**.** In this study, which includes papers published up to September 30, 2019, we are trying to refine the exposure to organic solvents and exclude other types of exposures, such as anaesthetic gases.

Organic solvents is mostly a mixture of hydrocarbons, including alcohols, aromatics, acetates, esters, halogenated hydrocarbons, ethers, glycols, ketones and petroleum distillates (Nelson et al. 1994), which makes it difficult to estimate the effect of single agents.

Highly exposed groups include floor layers, painters, dry cleaning workers and car painters. Among painters, the exposure in many places was high up to the 1980s when the exposure to paints with organic solvents was gradually replaced by water-based paints. Common organic solvents at that time were thinner, white spirits, trichloroethylene (degreasing agent) and tetrachloroethylene (dry cleaning and degreasing). Time-weighted average levels of tetrachloroethylene in dry cleaning during the 1970s could range between 20 and 150 mg/m^3^. (Lynge et al., 1997).

Among aromatic compounds, benzene (solvent in glues), styrene (lamination of reinforced plastics) and toluene (paint, printing) were common. Toluene, xylene, white spirits and other solvents in mixtures could be used in furniture and cabinet making, paint manufacture and painting, shoe manufacture, graphics industry and chemical industries.

A British study reported exposure levels of styrene for hand laminators between 170 and 430 mg/m^3^ during 1950–1980. A European study from reinforced plastic industries showed styrene exposures around 850 mg/m^3^ in the 1950s and 85 mg/m^3^ in the 1980s, that is a tenfold reduction (Lynge et al. 1997). Thus, there was a substantial decrease of the exposure to organic solvents from the late 1990s and onwards. As the effects are long term, however, earlier and higher exposures of organic solvents can have an impact decades later when the ageing process begins.

## Aims

The aim of the study was to perform a meta-analysis to investigate the relationship between the development of multiple sclerosis and work-related exposure to organic solvents.

## Methods

### Data source and search

The systematic literature search included PubMed, Scopus and the Cochrane library databases. References in the articles obtained were also carefully examined to find papers, which were not found in the systematic search. The search terms used were multiple sclerosis, workplace, employment, professions, career, occupations, occupational, organic solvents, case–control study, cohort study or epidemiol*.

### Inclusion criteria

After the removal of duplicates 406 articles remained. All titles and abstracts were scrutinized. The eligible criteria for being included in the meta-analysis were as follows:

1. Peer-reviewed studies in English, classified according to the Armon criteria (2003).
2. Human subjects with diagnosis of MS. Studies were excluded from the analysis if they did not list multiple sclerosis as an outcome.
3. Studies listing organic solvents as the dominating exposure source.
4. For inclusion in the meta-analysis, the studies had to supply sufficient data to calculate an odds ratio and an associated confidence interval.
5. All papers were reviewed independently by two physicians (LH and LG). If the classification differed, a consensus discussion followed to get a mutual agreement.

### Meta-analysis

**Fig. 1:**
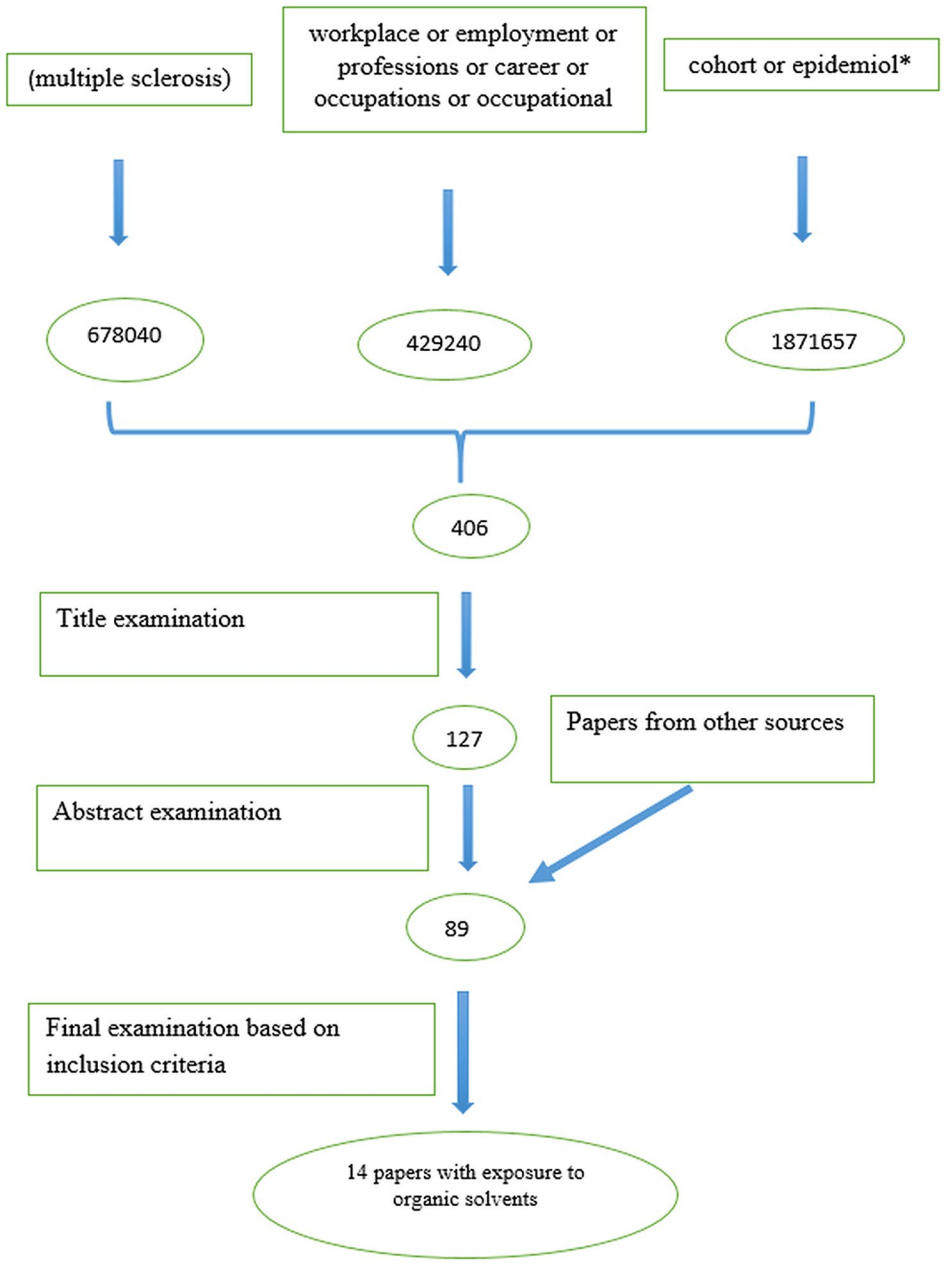
Flow chart for article search

**Fig. 2:**
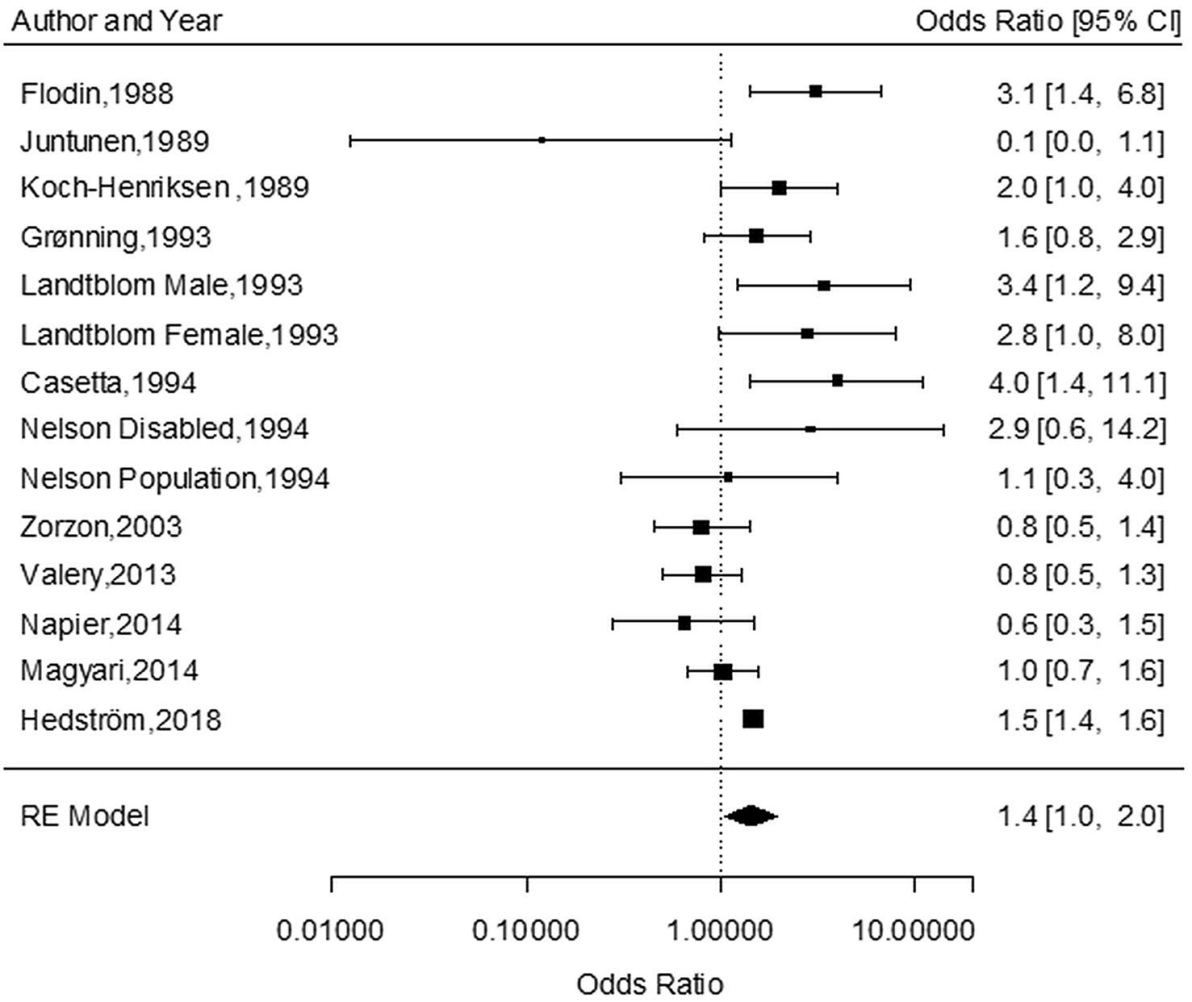
Meta-analysis of case–control studies

The online search identified 406 articles (Fig. 1). None of the publications fulfilled the Armon class I criteria (Armon 2003). Class II also includes studies of high quality. Even studies of class III can have a high scientific standard with an adequate control of bias and confounding. After the examinations of titles and abstracts 89 papers remained. After the final examination that was based on the inclusion criteria, 14 case–control studies fulfilling the Armon class II–III criteria remained for the meta-analysis. The risk estimates were reported as OR (Fig. 2). The outcome of two cohort studies was evaluated separately.

The meta-analysis calculates a weighed risk estimate for studies included. The weighed estimate takes into account the precision of the risk estimation for the individual studies.

A small study with a low precision (wide confidence interval) gets a lower weight, while a large study with higher precision gets a higher weight. The summary risk estimate is presented as an OR with a 95% confidence interval. *I*^2^-statistics with recommended cut-offs of 25, 50 and 75% showed that the heterogeneity among the studies was greater than if it had been due to chance. Accordingly, a random effects model was used.

The case–control studies included in this meta-analysis with total number of cases and referents as well as number of solvent-exposed cases and referents are presented in Table 1. Risk estimates with 95% confidence intervals, type of data collection and grading of the publications according to the Armon criteria (Armon 2003) are also presented.

**Table 1:** Case–control publications included in the meta-analysis with total number of cases and referents as well as number of solvent-exposed cases and referents

Publication	Total number of cases/references	Solvent-exposed cases/referents	OR 95% CI	Exposure	Armon criteria
Flodin et al. (1988)	83 vs 467	13 vs 39	3.1 (1.4–6.8)	Q + IIH	3
Juntunen (1989)	19 twin pairs	6 vs 1	0.1 (0.0–1.1)	ISOM	3
Koch-Henriksen (1989)	209 vs 209	40 vs 26	2.0 (1.0–4.0)	I	3
Grønning (1993)	155 vs 200	35 vs 29	1.6 (0.8–2.9)	Q + I	3
Landtblom (1993)	24 vs 172	14 vs 53	3.4 (1.2–9.4)	Q + TI	2
Landtblom (1993)	67 vs 176	10 vs 9	2.8 (0.9–8.0)	Q + TI	2
Casetta (1994)	104 vs 150	17 vs 7	4.0 (1.5–11.1)	Q + I	2
Nelson (1994)	299 vs 944	20 vs NP	2.9 (0.6–14.2)	IH	3
Nelson (1994)	299 vs 944	20 vs NP	1.1 (0.3–4.0)	IH	3
Zorzon (2003)	140 vs 131	27 vs 30	0.8 (0.5–1.4)	Q + I	3
Valery (2013)	276 vs 538	67 vs 137	0.8 (0.5–1.3)	Q + I	2
Napier (2014)	217 vs 496	10 vs 30	0.6 (0.3–1.5)	Q + I + TI	2
Magyari (2014)	1403 vs 25 ref/case	28 vs 638	1.0 (0.7–1.6)	DB + R	2
Hedström (2018)	2042 vs 2947	233 vs 232	1.5 (1.2–1.8)	Q	2

Risk estimates with 95% confidence intervals, type of data collection and grading according to the Armon criteria are also presented.

*Q* questionnaire, *I* interview, *TI* telephone interview, *IIH* interview by an industrial hygienist, *ISOM* interview by a specialist in occupational medicine, *DB + R* databases and registers, *NP* data not presented

Funnel plots were used to investigate a possible publication bias. If there is no publication bias, the OR estimates should be distributed symmetrically around the weighted OR. The Egger test and the Begg–Mazumdar test were used to further evaluate the funnel plot.

All analyses were performed using the R-package metafor (R version 3.4.2, R Core Team 2017): R: A language and environment for statistical computing; R Foundation for Statistical Computing, Vienna, Austria (URL https://www.R-pmroject.org/ (Viechtbauer 2010)).

## Results

As evident from Fig. 1, the online search identified 406 articles (Fig. 1). After title and abstract examinations 89 papers remained. After the final examination that was based on the inclusion criteria, 16 papers remained for the final meta-analysis (14 case–control studies and 2 cohort studies). The outcome of the meta-analysis of the 14 case–control studies is presented in Fig. 2 based on calculations by the R-package metafor.

The total random effects model results gave an Odds Ratio of 1.44 (95% CI 1.03–1.99) for the meta-analysis of the 14 case–control studies, which is a significant outcome (*p* = 0.02). The test for heterogeneity showed a *Q*-value (df = 14) of 34.64 with a *p* value of 0.001.

Besides the case–control studies, also two cohort studies were included in the material. Mortensen et al. (Mortensen et al. 1998) studied 124,766 subjects exposed to organic solvents and 87,501 unexposed controls. After 20 years of follow-up, 87 solvent-exposed males had developed MS compared to 90 in the general population and 94 in the comparison cohort. Riise et al. (Riise et al. 2002) followed a cohort of 11,542 painters from 1970 to 1986. During follow-up 9 painters received disability pension due to the development of MS (RR = 2.0; 95% CI 0.9–4.5). The age-adjusted relative risk was 1.9. No individual exposure data were available. In summary, the study by Mortensen et al. (Mortensen et al. 1998) was negative, while the meta-analysis by Riise et al. (Riise et al. 2002) indicated a slightly increased risk of developing MS.

When calculating Kendall’s tau as a measure of rank correlation for funnel plot asymmetry for the 14 case-controls studies, the tau-value from the Begg–Mazumdar’s test was 0.08 (*p* = 0.75). A similar result was obtained with the Egger’s test for funnel plot asymmetry showing a *p* value of 0.80. Accordingly, the outcome does not support the assumption of publication bias. Of the total model variation, 72.3% was attributable to the variation between the case–control studies and 27.7% was due to the variation within each study.

As evident from Fig. 2, the numerically larger case–control studies from 2003 to 2018 clearly show lower OR values as compared with the results presented by the smaller case–control studies reported during the late 1980s and 1990s. This trend was changed by the large case–control study presented by Hedstrom et al. (Hedstrom et al. 2018).

The case–control study by Juntunen (Juntunen et al. 1989) included 19 pair of twins (38 subjects). Of these, 21 subjects were diagnosed as multiple sclerosis (one subject each from 17 pair of twins and both subjects from two pair of twins). Six of 38 subjects had been exposed to organic solvents and another one for trichloroethylene. Of these, one subject had only a marginal exposure to organic solvents. Accordingly, six subjects were exposed. One of them had contracted multiple sclerosis but the other five had not. If excluding this study which has the widest confidence interval in the meta-analysis (see Fig. 2), the random effects model OR equals 1.50 (95% confidence limit 1.10–2.04; *p* = 0.011). The test of heterogeneity shows a *Q*-value (df = 12) of 29.95 and a *p* value of 0.003. The test of funnel plot asymmetry with the Begg–Mazumdar’s test gave a tau-value of 0.23 (*p* value 0.31). A similar outcome was shown by the Egger’s test giving a *p* value of 0.88. Thus, the outcome OR figures of the meta-analysis and the test results of the funnel plot asymmetry are more or less the same if the study by Juntunen et al. (Juntunen et al. 1989) is included or excluded from the meta-analysis.

As shown in Fig. 2, the odds ratios from the 14 case–control studies included in the meta-analysis ranged from 0.12 to 4.00. Among these, five studies presented a significant association between the development of multiple sclerosis and exposure to organic solvents (Flodin et al. 1988, Koch-Henriksen 1989; Landtblom et al. 1993; Casetta et al. 1994; Hedstrom et al. 2018). The strongest point estimate (OR = 4.0) was reported by Casetta et al. (Casetta et al. 1994). For the other four studies, the lower confidence limits were 1.4, 1.2 and 1.0, respectively. The results from the other nine case–control studies did not reach statistical significance (Fig. 2). One of the two cohort studies was negative and the other showed a tendency to an increased risk of developing MS.

## Discussion

Studies concerning the relationship between the development of multiple sclerosis and occupational exposure to organic solvents are rather few, and several studies are of limited sample size due to the rarity of the disease. This meta-analysis showed a slightly raised point estimate with a combined OR of 1.44 (1.03–1.99), which was marginally significant.

In the last decades there have been several systematic literature reviews trying to analyse the association between exposure to organic solvents and the development of MS. Landtblom et al. (Landtblom et al. 1996) conducted a review in 1996, showing relative risk point estimates that varied from 1.7 to 2.6, all significantly raised. Their results indicated that organic solvents might be a cause of multiple sclerosis.

In a review by Marrie (Marrie 2004) the aetiology of MS was assessed. Most of the case–control studies included in the meta-analysis used prevalent cases and relied on self-reported exposure to organic solvents. Adjustment for confounding, e.g. for gender or socioeconomic status, was often lacking, and some of the studies were based on small number. The conclusion by the author was that an association between multiple sclerosis and exposure to organic solvents could not be ruled out. Barragan-Martinez et al. (Barragan-Martinez et al. 2012) conducted a systematic review and meta-analysis in 2012 assessing organic solvents as a risk factor for autoimmune diseases. Fifteen studies were included in the meta-analysis giving an OR of 1.53 (95% CI 1.03–2.29). In this meta-analysis, however, also studies with exposure to anaesthetic gases were included.

The exposure to organic solvents was collected through questionnaires in some of the earlier studies in our review (Flodin et al. 1988; Gronning et al. 1993; Hedstrom et al. 2018, Koch-Henriksen 1989; Landtblom et al. 1993). Later, interviews based on questionnaires were introduced. Zorzon et al. (Zorzon et al. 2003) and Valery et al. (Valery et al. 2013) used face-to-face interviews that were based on questionnaires. Napier et al. (Napier et al. 2016) used telephone interviews to collect information through questionnaires. Another technique was applied by Casetta et al. (Casetta et al. 1994) who used five specially trained interviewers to complete a standardized questionnaire. Nelson et al. (Nelson et al. 1994) used information from personal records and industrial hygiene records to construct a database. Thereafter, two industrial hygienists constructed semi-quantitative exposure indices that were used to classify the exposure of the workers into different categories. The study by Magyari et al. (Magyari et al. 2014) was also register-based using different Danish population registers to classify the exposure from occupation titles. Recently, Hedstrom et al. (Hedstrom et al. 2018) published results from a Swedish population-based case–control study based on 2042 incident cases of MS and 2947 controls. Exposure to organic solvents increased the risk of MS (OR 1.5; 95% CI 1.2–1.8). In their model an interaction was seen between smoking, organic solvents, carriage of HLA-DRB1*15 and absence of HLA-A*02, which significantly increased the risk of developing MS. Exposure information was collected through a questionnaire where participants were asked whether they had been exposed to a number of items, including organic solvents, painting products, and varnish. In this study, about 75% of both cases and controls were females. If they reported previous exposure to organic solvents, painting products and/or varnish, they were classified as exposed, which is a rather rough exposure estimation since exposure intensity was not assessed.

In 1982, Amaducci et al. (Amaducci et al. 1982) published a paper based on a study of multiple sclerosis among shoe and leather workers in Italy, who were exposed to organic solvents from the glues used. Eighty-one patients with definitive or probable multiple sclerosis were interviewed. Of them, five patients were diagnosed with multiple sclerosis (two males and three females). Due to the low number of cases, however, this study was not included in our meta-analysis.

None of the studies have tried to measure the exposure by stationary or personal sampling, and thus, it was not possible to study a dose–response relationship. As described above, the way of estimating the exposure to organic solvents differed between the studies. Self-reporting of exposure is complicated if the exposure has taken place decades ago. In addition, work conditions have probably changed over time. MS is a rare disease and accordingly large sample sizes are needed to identify an effect. The long latency time between exposure and onset of symptoms makes it more difficult to investigate if the exposure precedes the outbreak of the disease (Marrie 2004).

In the Nordic countries, a high exposure to organic solvents took place from the 1950s–1980s. Some homogeneous occupational groups with often long exposure times include house painters, typographers/printers, and carpenters/cabinet makers. High solvent naphtha concentrations were measured when large surfaces were painted in poorly ventilated small rooms. Under such circumstances naphtha levels about 1800 mg/m^3^ were observed. When painting walls or ceilings with solvent-based paint an average level of 1260 mg/m^3^ was registered (Riala et al. 1984). During spray painting with alkyd paint, average solvent naphtha concentrations of 690 mg/m^3^ were noted. Rooms with mechanical ventilation had solvent concentrations less than one-fifth of the concentrations in rooms without ventilation. The use of mechanical respirators, however, decreased the exposure considerably. Overall, the study by Riala et al (1984) showed an average daily solvent naphtha exposure of about 240 mg/m^3^ for maintenance painters.

In Finland exposure levels from several surveys varied from 0.5 mg/m^3^ for benzene to 100 mg/m^3^ for styrene (Lynge et al. 1997). In surveys from Denmark the exposure to styrene was four times higher in the period 1955–1970 compared with 1981–1988 (Jensen et al. 1990). In a Swedish study of highly exposed rotogravure printers employed in 1925–1985, the exposure to average air levels of toluene ranged from a maximum of about 1720 mg/m^3^ in the 1940s and 1950s to about 115 mg/m^3^ by the mid-1980s (Svensson et al. 1990). Due to the high intake of organic solvents by inhalation, the uptake can be calculated by assuming a daily inhalation volume of 10 m^3^. Measurements in Finland showed urinary trichloroethylene concentrations in the mid-1960s about 100 µmol/L, compared to 40–50 µmol/L in the mid-1980s (Anttila et al. 1993). At the end of the 1980s–1990s, there was a dramatic reduction of the exposure to organic solvents when water-based products and improved work hygienic factors were enforced.

For some occupations, there can be a family tradition where the son continues with the same work as his father, e.g. among painters, car painters and shoemakers. However, the dominant factor behind symptoms and signs seems to be the exposure levels and the time of exposure. Genetic factors inherited from father to son that may give an increased sensitivity to solvent-induced effects on the CNS and PNS have been discussed, but so far the support for such a theory is limited.

The so-called funnel plots have for many years been used to detect small study effects.

One problem with this type of studies is the probability that a study will be included in a meta-analysis. Studies producing significant results are more likely to produce multiple publications and to be cited by other authors. Accordingly, positive studies will have a greater possibility to be included in a meta-analysis than negative studies, which may lead to bias (Sterne et al. 2000). The chance that studies with small sample sizes and low statistical precision will be published increases if they show stronger treatment effects (Gjerdevik and Heuch 2014).

**Fig. 3:**
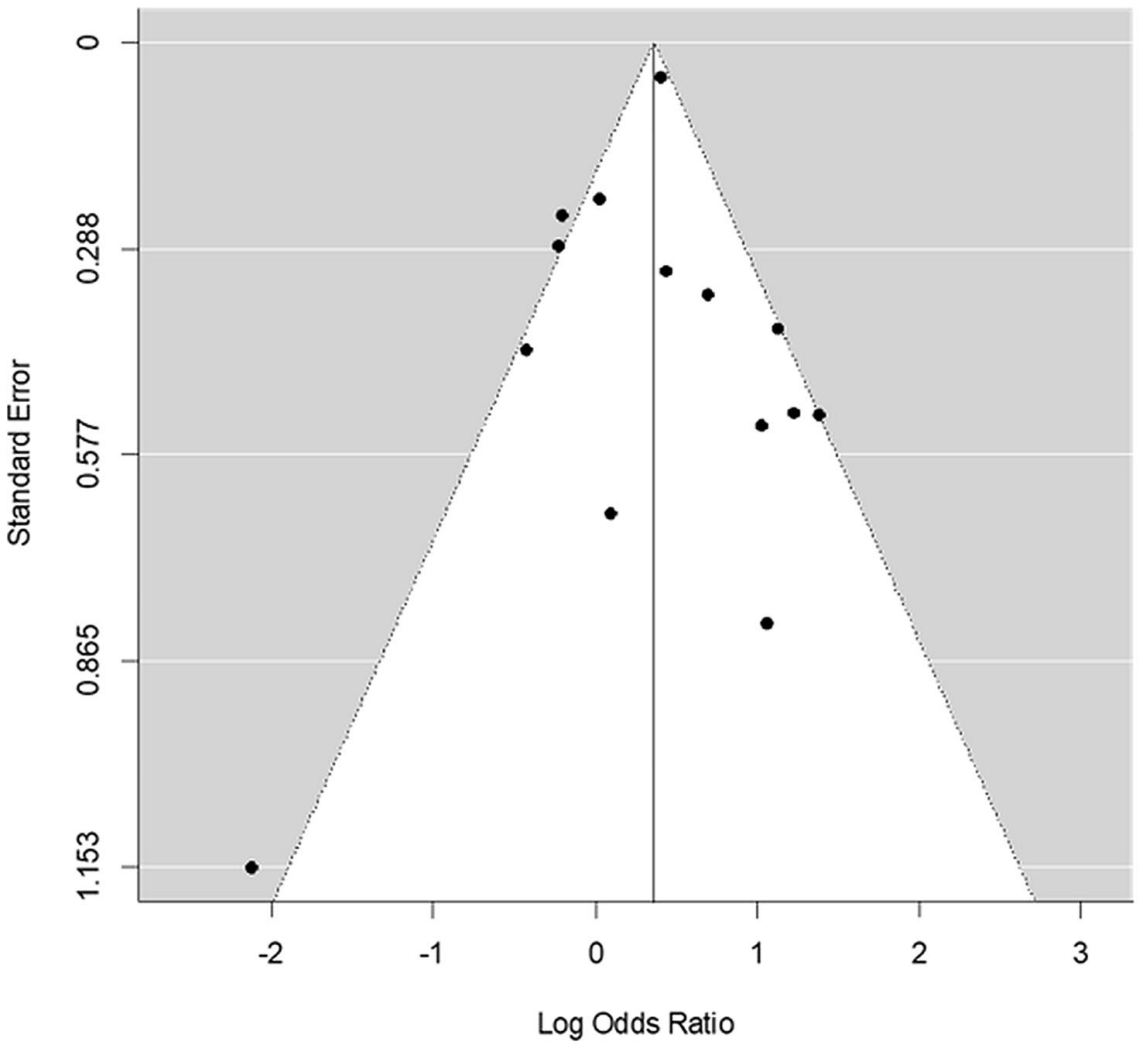
Funnel plot with approximately 95% confidence limits

Several tests have been used for detecting publication bias and small study effects. Egger et al. (Egger et al. 1997) used a simple linear regression of the effect estimate against its standard error, weighed by the inverse of the variance of the effect estimate. Begg and Mazumdar used the fact that publication bias will tend to induce a correlation between the treatment effects and their variance. The test examines the correlation between the two factors and standardizes the effect sizes prior to performing a rank correlation test based on Kendall’s tau (Gjerdevik and Heuch 2014). However, we found no indications of publication bias among the studies included in this meta-analysis (Fig. 3).

Heterogeneity is another way of introducing asymmetry in funnel plots. It is present when the true effects vary between the studies and is at hand if this variation is higher than could be expected by chance, which is the case in this meta-analysis.

In the future, longitudinal studies combining genetic, immunological and epidemiological markers are probably needed to understand the initiation of the disease and the trajectory of the disease progress. In years to come it may be possible to use endophenotypes to identify individuals at high risk and enroll them in interventional studies focusing on target environmental risk factors to prevent the disease from breaking out (Ramagopalan et al. 2010).

As MS is a complex multifactorial disease, it is important in future studies to include other risk factors when studying the causes of disease development. Recently, Hedstrom et al. (Hedstrom et al. 2018) presented a study, including genetic markers, smoking and exposure to organic solvents. A combined exposure to these three items increased the risk to develop MS considerably. The authors speculate that lung inflammation with a proinflammatory profile with concomitant exposure to tobacco smoking and organic solvents may interact with MS risk HLA genes and affect adaptive immunity.

The different summary OR results in this study, compared with some of the earlier reviews showing an increased risk, may partly be due to the fact that we have limited the exposure to organic solvents and excluded other exposures, e.g. anaesthetic gases. The earlier studies before the year 2000 was also smaller in size compared to later studies and collected information about exposure to organic solvents mainly by questionnaires. As found in our meta-analysis, smaller and earlier studies generally show larger effects than later and larger studies (the so-called small study effects). This meta-analysis is focused on work-related exposure to organic solvents. Exposure to organic solvents in leisure time is considered negligible in relation to the work-related exposure.

## Conclusion

The meta-analysis of the 14 case–control studies selected gave a summary OR of 1.44 (95% CI 1.03–1.99). The test of heterogeneity (*p* value 0.001) showed that the variability among these studies is greater than would be expected by chance. Four out of five larger studies published after 2003 did not reach statistical significance**.** Moreover, one of two cohort studies evaluated was negative, while the other showed a tendency to an increased risk of developing MS. The final interpretation of the result is that organic solvents may be slightly associated with an increased risk to develop MS. However, the relationship between MS and organic solvents is still not fully clarified. Also, other factors, such as smoking and genetic markers, may contribute and further increase the risk to develop the disease.
